# Enteral nutrition feeding in Chinese intensive care units: a cross-sectional study involving 116 hospitals

**DOI:** 10.1186/s13054-018-2159-x

**Published:** 2018-09-24

**Authors:** Juan Xing, Zhongheng Zhang, Lu Ke, Jing Zhou, Bingyu Qin, Hongkai Liang, Xiaomei Chen, Wenming Liu, Zhongmin Liu, Yuhang Ai, Difeng Wang, Qiuhui Wang, Qingshan Zhou, Fusen Zhang, Kejian Qian, Dongpo Jiang, Bin Zang, Yimin Li, Xiaobo Huang, Yan Qu, Yinguang Xie, Donglin Xu, Zhiqiang Zou, Xiangde Zheng, Jianbo Liu, Feng Guo, Yafeng Liang, Qiang Sun, Hongmei Gao, Yang Liu, Ping Chang, Aibin Ceng, Rongli Yang, Gaiqi Yao, Yun Sun, Xiaorong Wang, Yi Zhang, Yichao Wen, Jian Yu, Rongqing Sun, Zhiwei Li, Shiying Yuan, Yunlin Song, Peiyang Gao, Haiyan Liu, Zhaohui Zhang, Yunfu Wu, Biao Ma, Qiang Guo, Feng Shan, Mingshi Yang, Hailing Li, Yuanfei Li, Weihua Lu, Lei Wang, Chuangyun Qian, Zhiyong Wang, Jiandong Lin, Rumin Zhang, Peng Wan, Zhiyong Peng, Yuqiang Gong, Linxi Huang, Guobao Wu, Jie Sun, Yijun Deng, Dongwu Shi, Lixin Zhou, Fachun Zhou, Qindong Shi, Xiaodong Guo, Xueyan Liu, Weidong Wu, Xiangzhong Meng, Liandi Li, Weiwei Chen, Shusheng Li, Xianyao Wan, Zhixin Chao, An Zhang, Liming Gu, Wei Chen, Jinglan Wu, Lihua Zhou, Zhenhuan Zhang, Yibing Weng, Yongshun Feng, Chunli Yang, Yongjian Feng, Sumin Zhao, Fei Tong, Dong Hao, Hui Han, Baocai Fu, Chuanyong Gong, Zhiping Li, Kunlin Hu, Qiuye Kou, Han Zhang, Jie Liu, Chuming Fan, Xin Zhou, Xiumei Chen, Junli Sun, Xuejun Zhou, Bin Song, Cheng Sun, Liyun Zhao, Xinglu Dong, Linlin Zhang, Dafei Tong, Zhiguo Pan, Chuangjie Cai, Donghao Wang, Yingjun Dong, Yuanqi Gong, Zhisong Wu, Xinke Meng, Ping Wang, Weiqin Li

**Affiliations:** 1 Nanjing General Hospital of Nanjing Military Command, No.305 Zhongshan East Road, Nanjing, 210002 China; 2 Department of emergency medicine, Sir Run Run Shaw Hospital, Zhejiang University School of Medicine, Hangzhou, China; 3 Henan Provincial People’s Hospital, Zhengzhou, China; 4 Zhongshan People’s Hospital, Zhongshan, China; 5 Shandong University Qilu Hospital, Jinan, China; 6 Changzhou No.2 People’s Hospital affiliated to Nanjing Medical University, Nanjing, China; 7 Jilin University First Hospital, Changchun, China; 8 Xiangya Hospital Central South University, Changsha, China; 9 Guizhou Medical University affiliated hospital, Guiyang, China; 10 Wuxi People’s Hospital, Wuxi, China; 11 Hubei Provincial People’s Hospital, Wuhan, China; 12 Tai’an Central Hospital, Tai’an, China; 13 First Affiliated Hospital of Nanchang University, Nanchang, China; 14 Third Military Medical University Daping Hospital, Chongqing, China; 15 China Medical University Second Affiliated Hospital, Shenyang, China; 16 Guangzhou Medical University First Affiliated Hospital, Guangzhou, China; 17 Sichuan Provincial People’s Hospital, Chengdu, China; 18 Qingdao Municipal Hospital Group, Qingdao, China; 19 Jining First People’s Hospital, Jining, China; 20 Guangzhou First Municipal People’s Hospital, Guangzhou, China; 21 Xiehe Affiliated Hospital of Fujian Medical University, Fuzhou, China; 22 Dazhou Central Hospital, Dazhou, China; 23 Inner Mongolia People’s Hospital, Huhehaote, China; 24 Zhejiang University School of Medicine Sir Run Run Shaw Hospital, Hangzhou, China; 25 Qindao University Medical College Affiliated Yantai Yuhuangding Hospital, Qingdao, China; 26 Tianjing People’s Hospital, Tianjin, China; 27 Tianjing First Central Hospital, Tianjin, China; 28 Tangshan Gongren Hospital, Tangshan, China; 29 Southern Medical University Zhujiang Hospital, Guangzhou, China; 30 North China University of Science and Technology Affiliated Hospital, Tangshan, China; 31 Dalian Central Hospital, Dalian, China; 32 Peking University Third Hospital, Beijing, China; 33 Anhui Medical University Second Affiliated Hospital, Hefei, China; 34 Wenzhou Medical University First Affiliated Hospital, Wenzhou, China; 35 Shanxi Provincial People’s Hospital, Taiyuan, China; 36 Guangzhou Medical University Second Affiliated Hospital, Guangzhou, China; 37 Second Affiliated Hospital of Dalian Medical University, Dalian, China; 38 Zhengzhou University First Affiliated Hospital, Zhengzhou, China; 39 First People’s Hospital of Kunming, Kunming, China; 40 Union Hospital Affiliated to Tongji Medical College of Huanzhong University of Science and Technology, Wuhan, China; 41 Xinjiang Medical University Affiliated First Hospital, Wulumuqi, China; 42 Chengdu University of Traditional Chinese Medicine Affiliated Hospital, Chengdu, China; 43 First Affiliated Hospital of Anhui Medical University, Hefei, China; 44 Yichang Central People’s Hospital, Yichang, China; 45 Suzhou Municipal Hospital, Suzhou, China; 46 Jining Medical College Affiliated Hospital, Jining, China; 47 First Affiliated Hospital of Soochow University, Suzhou, China; 48 Qindao University Medical College Affiliated Hospital, Qindao, China; 49 Central South University Third Xiangya Hospital, Changsha, China; 50 401 Military Hospital of China, Qindao, China; 51 Changsha Central Hospital, Changsha, China; 52 Yijishan Hospital of Wannan Medical College, Wuhu, China; 53 Shanxi Medical University First Affiliated Hospital, Taiyuan, China; 54 Kuming Medical University First Affiliated Hospital, Kuming, China; 55 Hebei Medical University Third Affiliated Hospital, Shijiazhuang, China; 56 First Affiliated Hospital of Fujian Medical University, Fuzhou, China; 57 Zibo Central Hospital, Zibo, China; 58 First People’s Hospital of Yichang, Yichang, China; 59 Wuhan University Zhongnan Hospital, Wuhan, China; 60 Wenzhou Medical University Second Affiliated Hospital, Wenzhou, China; 61 Shantou University Medical College First Affiliated Hospital, Shantou, China; 62 Zhongnan University Xiangya Second Hospital, Changsha, China; 63 Yunnan Second People’s Hospital, Kunming, China; 64 Yancheng First People’s Hospital, Yancheng, China; 65 First People’s Hospital of Foshan, Foshan, China; 66 Chongqing Medical University First Affiliated Hospital, Chongqing, China; 67 Xi’an Jiao Tong University First Affiliated Hospital, Xi’an, China; 68 Beijing Wujing General Hospital, Beijing, China; 69 Shenzhen People’s Hospital, Shenzhen, China; 70 Shanxi Dayi Hospital of Shanxi Academy of Medical Science, Taiyuan, China; 71 254 Military Hospital of China, Tianjin, China; 72 Qingdao University Affiliated Hospital, Qingdao, China; 73 Linhai First People’s Hospital, Linhai, China; 74 Tongji Hospital of Tongji Medical College of Huazhong University of Science and Technology, Wuhan, China; 75 First Affiliated Hospital of Dalian Medical University, Dalian, China; 76 Beijing Chaoyang Hospital, Beijing, China; 77 Chongqing Medical University Second Affiliated Hospital, Chongqing, China; 78 People’s Hospital of Yuxi City, Yuxi, China; 79 Shijitan Hospital of Capital Medical University, Beijing, China; 80 Shenzhen Nanshan People’s Hospital, Shenzhen, China; 81 Affiliated Hospital of Inner Mongolia Medical College, Huhehaote, China; 82 Benq Hospital, Nanjing, China; 83 Luhe Hospital, Beijing, China; 84 Beijing Jingmei Group General Hospital, Beijing, China; 85 Jiangxi Provincial People’s Hospital, Nanchang, China; 86 Jinan University First Affiliated Hospital, Jinan, China; 87 General Hospital of Rocket Army, Beijing, China; 88 Hebei Medical University Second Affiliated Hospital, Shijiazhuang, China; 89 Binzhou Medical College Affiliated Hospital, Binzhou, China; 90 Chinese PLA General Hospital, Beijing, China; 91 Yantai Mountain Hospital, Yantai, China; 92 Tianjing Hospital of ITCWM Nankai Hospital, Tianjing, China; 93 Hunan Provincial People’s Hospital, Changsha, China; 94 People’s Hospital of Guangxi Zhuang Autonomous Region, Nanning, China; 95 Sun Yat-sen University Sixth Affiliated Hospital, Guangzhou, China; 96 China Academy of Chinese Medical Sciences Xiyuan Hospital, Beijing, China; 97 Wuhan General Hospital of Guangzhou Military Region, Wuhan, China; 98 First People’s Hospital of Yunnan, Kunming, China; 99 Xinjiang Military General Hospital, Wulumuqi, China; 100 Miyun Hospital of Beijing, Beijing, China; 101 Luoyang Central Hospital, Luoyang, China; 102 Huairou First Hospital of Beijing, Beijing, China; 103 Military General Hospital of Beijing PLA, Beijing, China; 104 Guangdong Provincial People’s Hospital, Guangzhou, China; 105 Guangdong Second TCM Hospital, Guangzhou, China; 106 Beijing Hospital of TCM, Beijing, China; 107 Anhui Provincial Hospital, Hefei, China; 108 Shenyang First People’s Hospital, Shenyang, China; 109 Guangzhou Military General Hospital, Guangzhou, China; 110 Sun Yat-sen University First Affiliated Hospital, Guangzhou, China; 111 Tianjing Cancer Hospital, Tianjing, China; 112 Shanxi Cancer Hospital, Taiyuan, China; 113 Nanchang University Second Affiliated Hospital, Nanchang, China; 114 Beijing University of Chinese Medicine Affiliated Dongfang Hospital, Beijing, China; 115 Shenzhen Second People’s Hospital, Shenzhen, China; 116 Chendu Fifth People’s Hospital, Chendu, China

**Keywords:** Enteral feeding, Intensive care units, Cross-sectional study

## Abstract

**Background:**

There is a lack of large-scale epidemiological data on the clinical practice of enteral nutrition (EN) feeding in China. This study aimed to provide such data on Chinese hospitals and to investigate factors associated with EN delivery.

**Methods:**

This cross-sectional study was launched in 118 intensive care units (ICUs) of 116 mainland hospitals and conducted on April 26, 2017. At 00:00 on April 26, all patients in these ICUs were included. Demographic and clinical variables of patients on April 25 were obtained. The dates of hospitalization, ICU admission and nutrition initiation were reviewed. The outcome status 28 days after the day of investigation was obtained.

**Results:**

A total of 1953 patients were included for analysis, including 1483 survivors and 312 nonsurvivors. The median study day was day 7 (IQR 2–19 days) after ICU entry. The proportions of subjects starting EN within 24, 48 and 72 h after ICU entry was 24.8% (84/352), 32.7% (150/459) and 40.0% (200/541), respectively. The proportion of subjects receiving > 80% estimated energy target within 24, 48, 72 h and 7 days after ICU entry was 10.5% (37/352), 10.9% (50/459), 11.8% (64/541) and 17.8% (162/910), respectively. Using acute gastrointestinal injury (AGI) 1 as the reference in a Cox model, patients with AGI 2–3 were associated with reduced likelihood of EN initiation (HR 0.46, 95% CI 0.353–0.599; *p* < 0.001). AGI 4 was significantly associated with lower hazard of EN administration (HR 0.056; 95% CI 0.008–0.398; *p* = 0.004). In a linear regression model, greater Sequential Organ Failure Assessment scores (coefficient – 0.002, 95% CI – 0.008 to − 0.001; *p* = 0.024) and male gender (coefficient – 0.144, 95% CI – 0.203 to − 0.085; *p* < 0.001) were found to be associated with lower EN proportion. As compared with AGI 1, AGI 2–3 was associated with lower EN proportion (coefficient – 0.206, 95% CI – 0.273 to − 0.139; *p* < 0.001).

**Conclusions:**

The study showed that EN delivery was suboptimal in Chinese ICUs. More attention should be paid to EN use in the early days after ICU admission.

## Background

Patients requiring intensive care unit (ICU) admission are at increased risk of death, owning to a variety of life-threatening conditions such as respiratory failure, shock, severe infection and multiple organ dysfunction [1, 2]. Metabolic response to critical illness is characterized by accelerated catabolism, resulting in wasting and negative nitrogen balance [3]. Nutritional support during the catabolic phase will not only lead to positive nitrogen balance but also prevent weakness and, eventually, multiple organ failure and death [4, 5]. Regarding the route of energy administration, enteral feeding has been proven to be superior to the parenteral nutrition in terms of the outcomes such as nosocomial infection, medical cost saving and even mortality rate [6–9]. Thus, enteral nutrition (EN) feeding is the primary choice of nutrition therapy in current clinical practice; a careful and well-monitored approach is proposed based on the risk of poor tolerance [10]. Critically ill patients usually have compromised gastrointestinal function, making it difficult to increase EN to a target. Other factors that can delay achieving a target EN proportion include compromised gastric function, abdominal surgery and unstable hemodynamics [11]. The clinical practices of EN feeding vary substantially across different regions and hospitals.

China has the world’s largest population of critically ill patients, and the clinical practice of EN feeding varies substantially across regions and hospitals. However, to the best of our knowledge, there is a lack of large-scale data on the practice of enteral nutrition in ICUs. Previous small studies have shown that the proportion of EN was as low as 40% on day 2, which could probably be improved with implementation of an EN feeding protocol [12, 13]. Compliance to the clinical practice guidelines was also found to be suboptimal in neurological ICUs [14]. Due to the lack of epidemiological data on clinical practice of EN in Chinese ICUs, a nationwide cross-sectional study was performed. The objective of the study was to provide epidemiological data on the clinical practice of EN feeding in Chinese ICUs. Also, factors associated with the initiation and proportion of EN were investigated. We hypothesized that the severity of illness and gastrointestinal function would influence the timing and quantity of EN feeding.

## Methods

### Study design

This cross-sectional study was conducted in 118 ICUs of 116 hospitals covering most provinces of mainland China on April 26, 2017. The types of ICUs in this study vary, including general ICUs, surgical ICUs, medical ICUs, respiratory ICUs, coronary ICUs, emergency ICUs and so on. Demographic and clinical variables of patients on April 25, 2017 (hereafter referred to as “the study day”) were recorded. The outcome of the study subjects on the 28th day after investigation was obtained. Data collection was performed via a customized website. The study was approved by the ethics committee of Jinling Hospital (approval no. 2017NZKY-010-01).

### Study population

At 00:00 on April 26, 2017, all patients in the study ICUs were included. Exclusion criteria were: patients or their surrogate refused to participate in the study; and patients whose 28-day outcomes were not recorded.

### Variables

Demographics such as age, gender, source of admission, weight (past weight and temporal weight) and height were obtained. The past weight was defined as the body weight when patients were healthy or before 1 week, whereas the temporal weight was the body weight on the study day or within 1 week. The dates of hospital admission and ICU admission were reviewed.

Routine laboratory tests of blood samples were measured, for example, C-reactive protein (CRP), percentage of lymphocyte, albumin, maximum and minimum blood glucose, and arterial lactate. The level of consciousness was estimated using the Glasgow Coma Scale (GCS). If a patient was under sedation, the sedative was reduced or discontinued to make an appropriate assessment. Vital signs such as mean blood pressure and some particular treatments such as using vasopressors were recorded for Sequential Organ Failure Assessment (SOFA) and Acute Physiologic and Chronic Health Evaluation II (APACHE II) scores. The maximum and minimum blood glucose (BG) values on the study day were also reported. If a patient had more than one measurement of these variables on the study day, the worst one was chosen.

The date for the start of EN was retrospectively recorded in the study, but details regarding EN delivery were only recorded on the study day. Gastrointestinal function was evaluated as normal or mild injured, moderate to severe injured and failure referring to the acute gastrointestinal injury (AGI) grading. Enteral feeding volume and energy density of EN were recorded in the units of milliliters and kilocalories per milliliter, respectively. The EN proportion of target was computed as the ratio of the energy provided by EN to the estimated energy target (= past body weight × 25 kcal/kg). The nutrition could be warmed and diluted, and the dilution effect was considered in calculating the targets–achievement ratio. The enteral feeding routes included gastric feeding, postpyloric feeding, percutaneous gastrostomy/jejunostomy (PEG/J), jejunostomy and others [15]. The EN administration styles included continuous pump, gravity, intermittent feeding and other styles. The body positions were recorded as elevation of the bed head by 30°, sitting or other positions. The frequency of gastric residual volume (GRV) measurement and the largest GRV were recorded. In a case when gastrointestinal decompression was used, GRV was not measured and the gastrointestinal decompression volume was measured. Abdominal pressure was estimated using the bladder pressure. Duration of EN feeding was defined as the hours of the study day during which EN was administered. The times and causes of EN discontinuation during the study day were recorded. Intolerance to EN included nausea, vomiting, aspiration, abdominal pain and distension, and diarrhea. The presence of nausea and vomiting were recorded as yes, no, unable to judge and so on. The severity of abdominal pain and distension were grade-evaluated. Stool frequency and total volume on the study day were recorded.

The outcome status at 28 days after the study day was recorded as the endpoint. The outcome variable was labeled as alive, dead and lost, corresponding to vital status of a subject. If a patient’s condition on day 28 was not available, he/she was considered lost to follow-up.

### Patients with missing outcomes

Missing values were present in the dataset. We performed single imputation for missing values [16]. For patients with missing values on outcome, their baseline characteristics were also reported and compared with those with a complete dataset.

### Statistical analysis

Continuous variables were expressed as mean and standard error, or median and interquartile range, as appropriate. Categorical data were expressed as the number and proportion. Because there were two groups in the study, analysis of variance was performed for normally distributed data and the Kruskal–Wallis test was used for nonnormal data. Categorical data were compared using the chi-square test [16, 17].

The initiation of EN was considered as survival data and a multivariable Cox proportional hazard regression model was performed to investigate factors associated with the initiation of EN [17]. Variables included in the model were the source of admission, age, SOFA and APACHE II scores, gender, AGI grading and GCS score. The selection of variables was based on the literature finding that severity of illness was associated with EN delivery, and demographics of age and gender were also included.

The factors associated with the proportion of EN on the study day were investigated in a multivariable linear regression model [18]. Variables were selected using a stepwise backward elimination method and the best model was judged by the Akaike information criterion (AIC) [19]. The final model included variables such as the source of admission, age, APACHE II score, SOFA score, gender, AGI grading and GCS score. The cross-sectional study was subject to length bias, in which patients who had a longer length of stay in the ICU were more likely to be included in the study. Thus, the patients included in the study were not an unbiased sample of the target population. To adjust for this length bias, we assigned higher weights to patients with shorter LOS in the ICU [20].

All statistical analyses were performed using R (version 3.3.2) [21]. Two-tailed *p* < 0.05 was considered to indicate statistical significance.

## Results

### Characteristics of enrolled patients

The baseline characteristics and variables for the study day are presented in Table 1. A total of 1953 patients were included for analysis, including 1483 survivors and 312 nonsurvivors. The median study day was day 7 (IQR 2–19 days) after ICU entry, and there was no difference between survivors and nonsurvivors. Nonsurvivors were significantly older than survivors (median 70 vs 66 years; *p* = 0.005 for two-group comparison). There were more males among both survivors (68%) and nonsurvivors (63%). Most of the study patients came from emergency departments (35%), followed by surgery (29%) and internal medicine (19%). The distribution of admission wards was similar for survivors and nonsurvivors. Past and temporal weight (median 65 vs 65 kg, 65 vs 63 kg; *p* = 0.369, 0.198 for two-group comparison) and EN start day (median 1 (0, 2.5) vs 1 (0, 2) days; *p* = 0.912 for two-group comparison) showed no difference between the two groups.

**Table 1:** Cross-sectional characteristics between groups

Variable	Total (*n* = 1953)	Survivors (*n* = 1483)	Nonsurvivors (*n* = 312)	Lost to follow up (*n* = 158)	*p* value
Study day with reference to ICU entry	7 (2, 19)	7 (2, 19)	6 (2, 15.5)	7 (3, 20)	0.131
Age	67 (51, 80)	66 (51, 80)	70 (57.75, 81)	69.5 (51, 81)	0.008
Sex (male, %)	1315 (0.67)	1011 (0.68)	197 (0.63)	107 (0.68)	0.216
Source of admission					
Emergency room	664 (0.35)	509 (0.35)	95 (0.32)	60 (0.39)	0.008
Surgery	545 (0.29)	430 (0.3)	73 (0.24)	42 (0.27)	0.008
Internal medicine	362 (0.19)	255 (0.18)	81 (0.27)	26 (0.17)	0.008
Other ward	126 (0.07)	96 (0.07)	16 (0.05)	14 (0.09)	0.008
Other hospital	202 (0.11)	155 (0.11)	36 (0.12)	11 (0.07)	0.008
Past weight	65 (60, 75)	65 (59.45, 75)	65 (58, 74)	67 (60, 75)	0.443
Temporal weight	65 (55, 70)	65 (55, 70)	63 (54.2, 70)	65 (60, 70)	0.263
Height	170 (160, 175)	170 (160, 175)	167 (160, 173)	170 (160, 175)	0.034
CRP (mmol/l)	44.6 (16.8, 95.95)	40.05 (15.5, 87)	74.1 (29.85, 139.75)	43.75 (14.3, 98.53)	0
Lymphocyte (%)	10.7 (6.3, 17.6)	11.1 (6.8, 18.4)	8 (5.07, 13.85)	10.1 (6.3, 16.7)	0
Albumin (mg/dl)	31.7 (27.8, 35.3)	32 (28.1, 35.6)	30 (26.2, 34.2)	32 (28.55, 35.35)	0
Maximum BG (mmol/l)	10.1 (8, 13.1)	10 (7.9, 12.9)	11.1 (8.75, 14.85)	11.3 (8.3, 13.8)	0
Minimum BG (mmol/l)	6.4 (5.4, 7.8)	6.3 (5.4, 7.6)	6.8 (5.6, 8)	6.05 (5.2, 7.9)	0.054
Lactate (mmol/l)	1.4 (1, 2)	1.3 (0.9, 1.9)	1.7 (1.2, 2.58)	1.4 (0.95, 1.85)	0
P/F ratio	260 (182, 348)	268 (196, 350)	227 (135, 320)	251 (160.5330)	0
Platelet (× 10^9^/l)	176 (115, 250)	184 (124, 260)	146 (82.25, 206.25)	173 (108.75, 257)	0
Total bilirubin (mmol/l)	12 (7, 19)	12 (7, 18)	14 (8, 23)	11 (8, 18.5)	0.001
Mean blood pressure (mmHg)	82 (72, 94)	83 (73, 94)	80 (70, 93)	81 (70.5, 94.5)	0.104
GCS score	12 (7, 15)	13 (7.75, 15)	9 (5, 15)	11 (6, 15)	0
Serum creatinine (mmol/l)	67 (48, 102.5)	64 (47, 94)	84 (55, 150)	79.5 (55, 181.25)	0
Urine output (ml/24 h)	1800 (1100, 2468)	1850 (1200, 2500)	1500 (770, 2322.5)	1800 (1007.5, 2400)	0
SOFA score	5 (3, 7)	5 (3, 7)	7 (5, 10)	6 (4, 8)	0
Body temperature (°C)	37 (36.7, 37.7)	37 (36.6, 37.6)	37.2 (36.7, 38)	37 (36.7, 37.52)	0.002
Heart rate (/min)	90 (80, 107)	90 (79, 105)	98 (85, 120)	91 (80, 105)	0
Respiratory rate (/min)	20 (17, 24)	20 (16, 24)	20 (18, 26)	20 (16, 23)	0.003
White blood cell (× 10^9^/l)	10 (7.2, 13.7)	9.8 (7.1, 13)	11 (7.75, 15.3)	10.2 (7.5, 14.55)	0.001
APACHE II score	17 (12, 22)	16 (11, 21)	20 (15, 26)	19 (13, 23)	0
EN start day	1 (0, 3)	1 (0, 2.5)	1 (0, 2)	1 (0, 3.75)	0.099
EN in prior 24 h	1270 (0.66)	966 (0.67)	206 (0.66)	98 (0.65)	0.951
AGI 1	1440 (0.86)	1141 (0.88)	214 (0.78)	85 (0.79)	0
AGI 2–3	210 (0.12)	142 (0.11)	51 (0.18)	17 (0.16)	0
AGI 4	33 (0.02)	16 (0.01)	11 (0.04)	6 (0.06)	0
Total volume of EN (ml)	1000 (800, 1250)	1000 (830, 1300)	1000 (600, 1080)	1000 (1000, 1225)	0.203
Energy density of EN (kcal/ml)	1 (1, 1.5)	1 (1, 1.5)	1 (1, 1.3)	1 (1, 1.5)	0.112
EN warming	53 (0.04)	37 (0.04)	11 (0.06)	5 (0.06)	0.426
EN dilution	538 (0.47)	414 (0.48)	87 (0.46)	37 (0.47)	0.944
EN route					
Gastric	1040 (0.85)	783 (0.84)	177 (0.88)	80 (0.91)	0.662
Jejunostomy	10 (0.01)	9 (0.01)	1 (0)	0 (0)	0.662
PEG/J	20 (0.02)	19 (0.02)	1 (0)	0 (0)	0.662
Postpyloric	127 (0.1)	101 (0.11)	19 (0.1)	7 (0.08)	0.662
Other	23 (0.02)	20 (0.02)	2 (0.01)	1 (0.01)	0.662
EN delivery method					
Continuous pump	1073 (0.88)	821 (0.89)	178 (0.88)	74 (0.85)	0.368
By gravity	35 (0.03)	27 (0.03)	6 (0.03)	2 (0.02)	0.368
Intermittent	86 (0.07)	59 (0.06)	17 (0.08)	10 (0.11)	0.368
Other	22 (0.02)	20 (0.02)	1 (0)	1 (0.01)	0.368
Position					
Bed head ≥30°	1004 (0.83)	771 (0.83)	164 (0.82)	69 (0.79)	0.899
Bed head < 30°	194 (0.16)	143 (0.15)	34 (0.17)	17 (0.2)	0.899
Sitting	11 (0.01)	9 (0.01)	1 (0)	1 (0.01)	0.899
Other	7 (0.01)	6 (0.01)	1 (0)	0 (0)	0.899
Frequency of GRV measurement	2 (0, 4)	3 (0, 4)	2 (0, 4)	3 (0, 6)	0.353
Maximum GRV (ml)	0 (0, 50)	0 (0, 50)	10 (0, 52.5)	10 (0, 50)	0.517
Use of gastrointestinal decompression	334 (0.17)	229 (0.16)	76 (0.25)	29 (0.18)	0.001
Gastrointestinal decompression (ml)	100 (40, 250)	100 (40, 200)	100 (50, 300)	100 (30, 300)	0.854
Abdominal pressure (mmHg)	11.36 (8.27, 14.19)	11.03 (8.82, 14)	12.5 (9.43, 15.33)	7.35 (5.88, 10)	0.083
Duration of EN delivery (hours)	20 (15, 24)	20 (14, 24)	21 (12, 24)	24 (20, 24)	0.001
EN discontinuation due to intolerance (times)					
0	997 (0.95)	775 (0.95)	161 (0.93)	61 (0.94)	0.318
1	31 (0.03)	21 (0.03)	6 (0.03)	4 (0.06)	0.318
2	20 (0.02)	15 (0.02)	5 (0.03)	0 (0)	0.318
3	2 (0)	1 (0)	1 (0.01)	0 (0)	0.318
4	2 (0)	1 (0)	1 (0.01)	0 (0)	0.318
5	1 (0)	1 (0)	0 (0)	0 (0)	0.318
EN discontinuation due to examination (times)					
0	950 (0.9)	732 (0.9)	156 (0.91)	62 (0.95)	0.13
1	84 (0.08)	68 (0.08)	14 (0.08)	2 (0.03)	0.13
2	11 (0.01)	11 (0.01)	0 (0)	0 (0)	0.13
3	1 (0)	0 (0)	0 (0)	1 (0.02)	0.13
4	1 (0)	1 (0)	0 (0)	0 (0)	0.13
5	2 (0)	1 (0)	1 (0.01)	0 (0)	0.13
6	2 (0)	1 (0)	1 (0.01)	0 (0)	0.13
EN discontinuation due to other reasons (times)					
0	983 (0.95)	753 (0.94)	166 (0.96)	64 (0.98)	0.99
1	32 (0.03)	27 (0.03)	4 (0.02)	1 (0.02)	0.99
2	8 (0.01)	7 (0.01)	1 (0.01)	0 (0)	0.99
3	4 (0)	3 (0)	1 (0.01)	0 (0)	0.99
5	6 (0.01)	5 (0.01)	1 (0.01)	0 (0)	0.99
6	3 (0)	3 (0)	0 (0)	0 (0)	0.99
Presence of nausea					
No	1670 (0.86)	1303 (0.88)	260 (0.84)	107 (0.69)	0.000
Unable to judge	176 (0.09)	107 (0.07)	32 (0.1)	37 (0.24)	0.000
Yes	93 (0.05)	63 (0.04)	18 (0.06)	12 (0.08)	0.000
Presence of vomiting					
No	1743 (0.9)	1338 (0.91)	272 (0.87)	133 (0.85)	0.108
Suspected	65 (0.03)	44 (0.03)	12 (0.04)	9 (0.06)	0.108
Unable to judge	29 (0.01)	21 (0.01)	5 (0.02)	3 (0.02)	0.108
Yes	105 (0.05)	70 (0.05)	23 (0.07)	12 (0.08)	0.108
Presence of aspiration					
No	1809 (0.94)	1378 (0.94)	296 (0.95)	135 (0.87)	0.008
Suspected	87 (0.05)	59 (0.04)	11 (0.04)	17 (0.11)	0.008
Unable to judge	20 (0.01)	17 (0.01)	1 (0)	2 (0.01)	0.008
Yes	17 (0.01)	12 (0.01)	4 (0.01)	1 (0.01)	0.008
Presence of abdominal pain					
No	1436 (0.74)	1133 (0.77)	214 (0.69)	89 (0.57)	0
Persistent	77 (0.04)	51 (0.03)	18 (0.06)	8 (0.05)	0
Self resolution	93 (0.05)	66 (0.04)	15 (0.05)	12 (0.08)	0
Unable to judge	329 (0.17)	218 (0.15)	63 (0.2)	48 (0.31)	0
Presence of abdominal distension					
Mild	420 (0.22)	311 (0.21)	66 (0.21)	43 (0.27)	0.000
No	1347 (0.69)	1055 (0.71)	199 (0.64)	93 (0.59)	0.000
Obvious	100 (0.05)	60 (0.04)	28 (0.09)	12 (0.08)	0.000
Severe	18 (0.01)	11 (0.01)	6 (0.02)	1 (0.01)	0.000
Unable to judge	62 (0.03)	40 (0.03)	13 (0.04)	9 (0.06)	0.000
Bowel sound					
Hyper	37 (0.02)	27 (0.02)	7 (0.02)	3 (0.02)	0
Hypo	443 (0.23)	295 (0.2)	103 (0.33)	45 (0.29)	0
None	125 (0.06)	90 (0.06)	22 (0.07)	13 (0.08)	0
Normal	1321 (0.69)	1047 (0.72)	179 (0.58)	95 (0.61)	0
Stool					
Yes	1103 (0.58)	840 (0.59)	175 (0.57)	88 (0.57)	0.469
No	579 (0.31)	436 (0.31)	98 (0.32)	45 (0.29)	0.469
No within 3 days	171 (0.09)	119 (0.08)	33 (0.11)	19 (0.12)	0.469
Colostomy	23 (0.01)	19 (0.01)	1 (0)	3 (0.02)	0.469
Incontinence	16 (0.01)	14 (0.01)	2 (0.01)	0 (0)	0.469
Stool frequency	1 (1, 2)	1 (1, 2)	1 (1, 2)	2 (1, 2)	0.943
Stool volume (ml)	200 (100, 350)	200 (100, 350)	200 (100, 300)	200 (100, 350)	0.266
Stool description					
Granular hard	18 (0.02)	15 (0.02)	2 (0.01)	1 (0.01)	0.946
Loose	528 (0.52)	400 (0.52)	92 (0.56)	36 (0.52)	0.946
Shaped	2 (0)	2 (0)	0 (0)	0 (0)	0.946
Shaped soft	412 (0.41)	323 (0.42)	61 (0.37)	28 (0.41)	0.946
Soft	5 (0)	4 (0.01)	1 (0.01)	0 (0)	0.946
Watery	45 (0.04)	32 (0.04)	9 (0.05)	4 (0.06)	0.946
Stool blood					
Black stool	16 (0.02)	9 (0.01)	7 (0.04)	0 (0)	0.174
Bloody stool	5 (0)	4 (0.01)	1 (0.01)	0 (0)	0.174
No	946 (0.94)	731 (0.95)	148 (0.91)	67 (0.96)	0.174
OB positive	38 (0.04)	28 (0.04)	7 (0.04)	3 (0.04)	0.174

Statistics expressed as number of patients (proportion) or median (first and third interquartile range)

*AGI* acute gastrointestinal injury, *BG* blood glucose, *APACHE II* Acute Physiology and Chronic Health Evaluation II, *CRP* C-reactive protein, *EN* enteral nutrition, *GCS* Glasgow Coma Score, *GRV* gastric residual volume, *ICU* intensive care unit, *PEG/J* percutaneous endoscopic gastrostomy/jejunostomy, *SOFA* Sequential Organ Failure Assessment, P/F parital pressure of oxygen/Fraction of inspired oxygen

For laboratory variables, CRP (median 40.1 vs 74.1 mg/dl; *p* < 0.001), maximum BG (median 10 vs 11.1 mmol/l; *p* < 0.001), minimum BG (median 6.3 vs 6.8 mmol/l; *p* < 0.001), lactate (median 1.3 vs 1.7 mmol/l; *p* < 0.001) and total bilirubin (median 12 vs 14 mmol/l; *p* < 0.001) were significantly lower in survivors than those in nonsurvivors, while the GCS was significantly higher (median 13 vs 9; *p* < 0.001). As expected, the SOFA score (median 7 vs 5; *p* < 0.001) and APACHE II score (median 20 vs 16; *p* < 0.001) were higher in the nonsurvivors than that in the survivors.

### EN delivery

There were 1440 patients in AGI 1 (86%), 210 patients in AGI 2–3 (12%) and 33 patients in AGI 4 (2%). The severity of AGI was associated with mortality, as there were significantly more patients with AGI 1 in the survivors (88% vs 78%; *p* < 0.001) than in the nonsurvivors, and more patients had AGI 2–3 (18% vs 11%; *p* = 0.001) and AGI 4 (4% vs 1%) in the nonsurvivors than in the survivors.

**Fig. 1:**
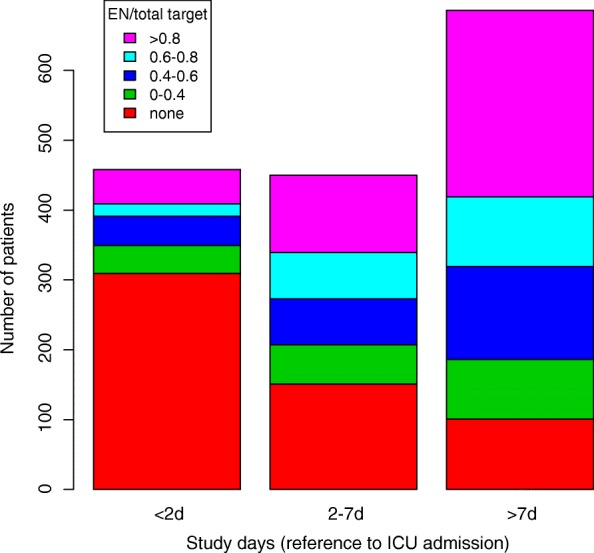
Number of patients with different EN proportions stratified by different study days. Nearly two thirds of patients did not use EN within 2 days after ICU entry. Both the proportion of patients using EN and EN proportions of total energy target increased over time, but less than half of patients received > 60% total target after first week in ICU. d days, EN enteral nutrition, ICU intensive care unit

There were 1270 patients using EN (66%) on the study day and there was no difference between survivors and nonsurvivors (67% vs 66%). The mean total volume of EN was 1000 ml in the two groups. The energy density of EN was 1 or 1.5 kcal/ml for different formulas. However, the proportion of starting EN within 24, 48 and 72 h after ICU entry was 23.9% (84/352), 32.7% (150/459) and 37.0% (200/541), respectively. The proportion of subjects receiving > 80% estimated energy target within 24, 48, 72 h and 7 days after ICU entry was 10.5% (37/352), 10.9% (50/459), 11.8% (64/541) and 17.8% (162/910), respectively. Data on the proportion of EN were only available for the study day. Thus, the calculation of EN proportion within 24, 48, 72 and 7 days only included patients for whom the study days were within the relevant time window (a subset of all patients) and not from all recruited patients. Figure 1 plots the number of patients with different EN proportions against the study days (e.g., with reference to the day of ICU entry), showing that the use of EN increased over time.

Gastric feeding was used in 85% patients, followed by postpyloric feeding (11%), PEG/J (2%) and jejunostomy (2%). Continuous pump (88%) was the predominant EN infusion style, followed by intermittent pump (7%) and infusion by gravity (3%). The majority of patients (83%) had their bed head elevated more than 30°. Warming was used in 47% of patients and EN was not diluted for the majority of cases (96%). The frequency and the total volume of GRV measurement were 2 (IQR 0–4) times and 0 (IQR 0–50) ml. The gastrointestinal depression volume and abdominal pressure were 100 (IQR 40–250) ml and about 14 (IQR 11–18) mmHg. EN was not discontinued for the majority of patients in the study day; the reasons for EN discontinuation were intolerance (52 patients, 5%), examination (98 patients, 9%) and others (52 patients, 5%). However, none of these EN-related parameters was significantly associated with mortality outcome, except for the proportion of gastrointestinal decompression (16% vs 25%; *p* < 0.001).

### Factors associated with EN initiation

In the Cox proportional hazard model (Table 2), we found that the AGI and GCS were associated with initiation of EN. Using AGI 1 as the reference, patients with AGI 2–3 were associated with reduced likelihood of EN initiation (HR 0.46, 95% CI 0.353–0.599; *p* < 0.001). AGI 4 was significantly associated with lower hazard of EN administration (HR 0.056, 95% CI 0.008–0.398; *p* = 0.004). Although a greater value of GCS was statistically associated with reduced likelihood of EN initiation, the clinical significance was marginal (HR 0.945, 95% CI 0.921–0.969; *p* < 0.001).

**Table 2:** Variables associated with enteral nutrition initiation

		HR	2.5%	97.5%	*p* value
Source of admission (emergency room as reference)	Surgery	1.087	0.878	1.346	0.443
	Internal medicine	1.166	0.935	1.455	0.174
	Other ward	1.125	0.701	1.805	0.626
	Other hospital	1.164	0.896	1.511	0.255
Age		1.002	0.997	1.007	0.371
APACHE II score		1.007	0.989	1.024	0.459
SOFA score		1.007	0.979	1.035	0.651
Sex (female as reference)		1.087	0.910	1.299	0.356
AGI (AGI 1 as reference)	AGI 2–3	0.460	0.353	0.599	< 0.001
	AGI 4	0.056	0.008	0.398	0.004
GCS score		0.945	0.921	0.969	< 0.001

*AGI* acute gastrointestinal injury, *APACHE II* Acute Physiology and Chronic Health Evaluation II, *GCS* Glasgow Coma Score, *HR* hazard ratio, *SOFA* Sequential Organ Failure Assessment

### Factors associated with proportion of EN delivery

In the multivariable linear regression model investigating factors associated with the EN proportion (Table 3), greater SOFA scores (coefficient – 0.002, 95% CI – 0.008 to − 0.001; *p* = 0.024) and male gender (coefficient – 0.144, 95% CI – 0.203 to − 0.085; *p* < 0.001) were found to be associated with lower EN proportion. As compared with AGI 1, AGI 2–3 was associated with lower EN proportion (coefficient – 0.206, 95% CI – 0.273 to − 0.139; *p* < 0.001).

**Table 3:** Variables associated with proportion of EN on the study day

		Coefficient	2.5%	97.5%	*p* value
Age		– 0.002	− 0.003	− 0.001	0.039
APACHE II score		0.004	− 0.001	0.009	0.114
SOFA score		− 0.002	− 0.008	− 0.001	0.024
Sex (female as reference)		− 0.144	− 0.203	− 0.085	0.000
Source of admission (emergency room as reference)	Internal medicine	0.036	− 0.101	0.173	0.610
	Other hospital	0.040	− 0.036	0.116	0.307
	Surgery	0.023	− 0.041	0.088	0.478
	Ward	− 0.026	− 0.094	0.043	0.464
EN start time (1-day increase)		− 0.006	− 0.006	− 0.012	0.001
AGI (AGI 1 as reference)	AGI 2–3	− 0.206	− 0.273	− 0.139	0.000
	AGI 4	− 0.370	− 1.234	0.495	0.401

*AGI* acute gastrointestinal injury, *APACHE II* Acute Physiology and Chronic Health Evaluation II, *EN* enteral nutrition, *SOFA* Sequential Organ Failure Assessment

## Discussion

This was the largest cross-sectional study in mainland China covering 118 ICUs in 116 hospitals. The study enrolled 1953 patients, including 1483 survivors and 312 nonsurvivors. The results showed that EN delivery was suboptimal in China, evidenced by only 32.7% of critically ill patients received EN feeding within 48 h after ICU admission. Only a minority of patients (17.8%) received 80% of the estimated energy target by EN within 7 days after ICU entry. In the multivariable analysis, AGI was independently associated with the initiation of EN, indicating that gastrointestinal dysfunction was the major obstacle prohibiting EN delivery. The severity of illness as reflected by the SOFA score was negatively associated with the EN proportion.

In a worldwide study involving 46 countries and 880 units, enteral feeding was prescribed to only 10% of patients on the first day but this number increased to more than 40% of patients after 5 days [22]. In our study, 23.9% patients received EN on the first day and this percentage increased to 37% within 3 days. In another study conducted in Latin America, 59.7% of patients received > 90% estimated daily target within 24 h after ICU admission [23]. The study included parenteral nutrition, enteral nutrition or a combination, and thus the proportion was much higher than that in our study. The time to achieve > 80% estimated energy target was longer in the present study as compared with other reports. For example, Yip et al. [24] reported that the mean time to achieve > 80% target was 1.8 days (SD 1.5 days). However, the proportion of patients receiving > 80% energy target in the first 2 days was only 10.9% (50/459) in our study. Meanwhile, the median volume of EN in our study was 1000 exactly, suggesting that a more standard 1 L per 24 h is used and not related to target or body weight or BMI. This seems more cultural, nonprotocolized. According to the recent clinical practice guidelines [25], EN should be initiated within 24–48 h for critically ill patients who are unable to maintain volitional intake. However, this target is far from being reached in most Chinese hospitals. A cohort study from China reported that the EN feeding protocol was able to increase the proportion of EN on day 2 (41.8 ± 22.3% vs 50.0 ± 28.3%; *p* = 0.006) [13]. Thus, every effort should be made to standardize the EN feeding protocol to prompt EN delivery for critically ill patients.

Factors associated with EN initiation were investigated in the study. The results showed that patients with advanced AGI stage were less likely to receive EN, which was consistent with other reports. In a multicenter study, AGI grading was found to be associated with EN intolerance and the discontinuation of EN feeding [26]. Although our study is cross-sectional in design and cannot deduce the causal relationship between AGI grade and EN discontinuation, we could propose that EN discontinuation might be attributable to the compromised gastrointestinal function. However, this practice may not be supported by empirical evidence, because there is evidence showing that advanced AGI grade should not be a contraindication of EN feeding. On the contrary, Jin et al. [27] showed that the AGI grade was improved in the early EN group (*F*-statistic = 4.468; *p* < 0.05), indicating that EN delivery for patients with moderate gastrointestinal impairment may help to accelerate the recovery of gastrointestinal function. Actually, any association between higher AGI and/or higher SOFA score and lower EN given indicates that increasing severity of disease is associated with decreasing EN tolerance as well as poorer outcome. It was counterintuitive to see that a greater value of GCS was statistically associated with reduced likelihood of EN initiation, but the clinical significance was marginal (HR 0.945, 95% CI 0.921–0.969; *p* < 0.001). This could be explained by the fact that for patients with low GCS caused by trauma or stroke, we usually insert a GI tube on admission and start EN very early. After all, gastrointestinal dysfunction or shock, one of the major factors limiting EN use, is less common in stroke or traumatic brain injury patients.

In a linear regression model, we investigated factors associated with the proportion of the estimated energy requirement delivered via EN. The results showed that the SOFA score, gender and AGI were independently associated with the proportion. No wonder that a higher SOFA score is an obstacle of EN increments, as it is a measurement of organ dysfunction for critically ill patients and circulatory failure is an important component of the total score [28]. The guidelines also suggest a delay of EN delivery in patients with circulatory failure [25]. A novel finding in the study was that male patients were less likely to reach the estimated energy target as compared to females. Some may argue that this can be a false positive finding due to multiple testing. However, the effect size was large with male patients 26% lower in proportion than female patients (*p* < 0.001). Such a large effect size cannot be simply explained by random errors [29]. Probably, the physicians were more likely to give a fixed dose to patients without considering their body weight. Since men’s body weight is usually greater than women’s, they might receive a lower proportion of EN than female patients. In the linear model, AGI appeared to be an independent predictor of EN proportion. It is reasonable that physicians reduce the amount of EN in the presence of signs and symptoms that exaggerate the AGI grading [30]. According to current guidelines, AGI was not a factor prohibiting the initiation of EN. For a patient with greater AGI, physicians can initiate but maybe not achieve the target so easily. Initiation has to do with protocols, awareness of the importance, preserving gut function and so on. Furthermore, the study showed that GI intolerance was a very uncommon reason to interrupt EN. Thus, the problem is not AGI but the culture of not starting EN.

Several limitations must be acknowledged. First, the study was a cross-sectional study that causal inference cannot be confirmed. For example, we could only conclude that there was an association between AGI and EN delivery, the causal relation cannot be well defined. While a high AGI score may prohibit physicians to add EN, it is also possible that EN delivery exacerbates the already compromised gastrointestinal function. Second, the study only recorded data on the study day and the EN delivery and gastrointestinal function before and after the study day remain unknown, which precluded the analysis of a temporal trend of the EN delivery. However, since we have a large number of patients, and the study day involved all consecutive days after ICU admission, this gives us an opportunity to investigate the current trend of EN delivery.

## Conclusion

The study showed that EN delivery was suboptimal in Chinese ICUs, and that EN use in the early days after ICU admission should be paid more attention. One reason for this is that most Chinese hospitals lack a standardized EN feeding protocol for critically ill patients.

## Abbreviations

AGI: Acute gastrointestinal injury
APACHE II: Acute Physiology and Chronic Health Evaluation II
BG: Blood glucose
CI: Confidence interval
CRP: C-reactive protein
EN: Enteral nutrition
GCS: Glasgow Coma Score
GRV: Gastric residual volume
HR: Hazard ratio
ICU: Intensive care unit
IQR: Interquartile range
PEG/J: Percutaneous endoscopic gastrostomy/jejunostomy
SOFA: Sequential Organ Failure Assessment
